# Inflammatory Protein Profiles in Plasma of Candidaemia Patients and the Contribution of Host Genetics to Their Variability

**DOI:** 10.3389/fimmu.2021.662171

**Published:** 2021-08-26

**Authors:** Vasiliki Matzaraki, Kieu T. T. Le, Martin Jaeger, Raúl Aguirre-Gamboa, Melissa D. Johnson, Serena Sanna, Diletta Rosati, Lude Franke, Alexandra Zhernakova, Jingyuan Fu, Sebo Withoff, Iris Jonkers, Yang Li, Leo A. B. Joosten, Mihai G. Netea, Cisca Wijmenga, Vinod Kumar

**Affiliations:** ^1^Department of Internal Medicine and Radboud Center for Infectious Diseases, Radboud University Medical Center, Nijmegen, Netherlands; ^2^Department of Genetics, University Medical Center Groningen, University of Groningen, Groningen, Netherlands; ^3^Division of Infectious Diseases, Duke University Medical Center, Durham, NC, United States; ^4^Department of Pediatrics, University Medical Center Groningen, University of Groningen, Groningen, Netherlands; ^5^Department for Genomics and Immunoregulation, Life and Medical Sciences Institute (LIMES), University of Bonn, Bonn, Germany; ^6^Department of Immunology, Kristian Gerhard (K.G). Jebsen Coeliac Disease Research Centre, University of Oslo, Oslo, Norway; ^7^Nitte University Centre for Science Education and Research (NUCSER), Nitte (Deemed to Be University), Deralakatte, India

**Keywords:** inflammatory proteins, protein-QTLs, *C. albicans*, candidaemia, survival, MMP-1, uPA

## Abstract

Circulatory inflammatory proteins play a significant role in anti-*Candida* host immune defence. However, little is known about the genetic variation that contributes to the variability of inflammatory responses in response to *C. albicans*. To systematically characterize inflammatory responses in *Candida* infection, we profiled 91 circulatory inflammatory proteins in peripheral blood mononuclear cells (PBMCs) stimulated with *C. albicans* yeast isolated from 378 individuals of European origin from the 500 Functional Genomics (500FG) cohort of the Human Functional Genomics Project (HFGP) and Lifelines Deep cohort. To identify the genetic factors that determine variation in inflammatory protein responses, we correlated genome-wide single nucleotide polymorphism (SNP) genotypes with protein abundance (protein quantitative trait loci, pQTLs) produced by the *Candida*-stimulated PBMCs. Furthermore, we investigated whether differences in survival of candidaemia patients can be explained by modulating levels of inflammatory proteins. We identified five genome-wide significant pQTLs that modulate IL-8, MCP-2, MMP-1, and CCL3 in response to *C. albicans*. In addition, our genetic analysis suggested that *GADD45G* from rs10114707 locus that reached genome-wide significance could be a potential core gene that regulates a cytokine network upon *Candida* infection. Last but not least, we observed that a trans-pQTL marked from SNP rs7651677 at chromosome 3 that influences urokinase plasminogen activator (uPA) is strongly associated with patient survival (*P*_survival_ = 3.52 x 10^-5^, OR 3). Overall, our genetic analysis showed that genetic variation determines the abundance of circulatory proteins in response to *Candida* infection.

## Introduction

*Candida* species are by far the most common fungal pathogens that cause both invasive and mucosal fungal infections. They have been described as the fourth most common cause of nosocomial bloodstream infection in the United States (1, 2). Invasive candidiasis causes more than 250,000 new systemic infections on a yearly basis and leads to more than 50,000 deaths (3). Most humans are colonized with *C. albicans* shortly after birth, which remains as part of a normal human’s microbiota. Infection occurs only if the epithelial barrier function is impaired and/or there are microbiome imbalances and/or the host immune system is compromised. Under these conditions, *Candida* can invade tissue and reach blood circulation. The bloodstream carries *Candida* to almost all vital organs, leading to systemic infections and, eventually, to organ failure followed by death.

Protective immunity to *Candida* involves both innate and adaptive cellular and humoral responses (4, 5). Cytokines and chemokines are a group of low molecular weight proteins that contribute significantly to anti-*Candida* host immune defence by acting as mediators between immune and non-immune cells, by enhancing the antifungal activity of immune cells and by attracting inflammatory immune cells to the site of infection. Earlier studies have shown the capacity of *C. albicans* to induce production of various cytokines and chemokines (6–9). However, all previous studies focused on a narrow spectrum of inflammatory proteins, and a systematic study of inflammatory proteins released in the blood circulation upon *C. albicans* infection is lacking. Given that proteins are inherently influenced by genetic factors, it is important to assess the impact of host genetics on *Candida*-induced inflammatory proteins. By studying the genetics of only six different cytokines upon *Candida*-stimulation, we showed that host genetics play a major role in inter-individual variability in cytokine responses, and single nucleotide polymorphisms (SNPs) affecting cytokine responses are associated with susceptibility to candidaemia (9–11). Given that disease prognosis of candidaemia patients is generally poor, and survival rates differ greatly among them (12), we also assumed that genetic variation determines patient survival. Therefore, we hypothesized that modulation of circulatory inflammatory proteins may determine the differences in survival among patients.

**Figure 1:**
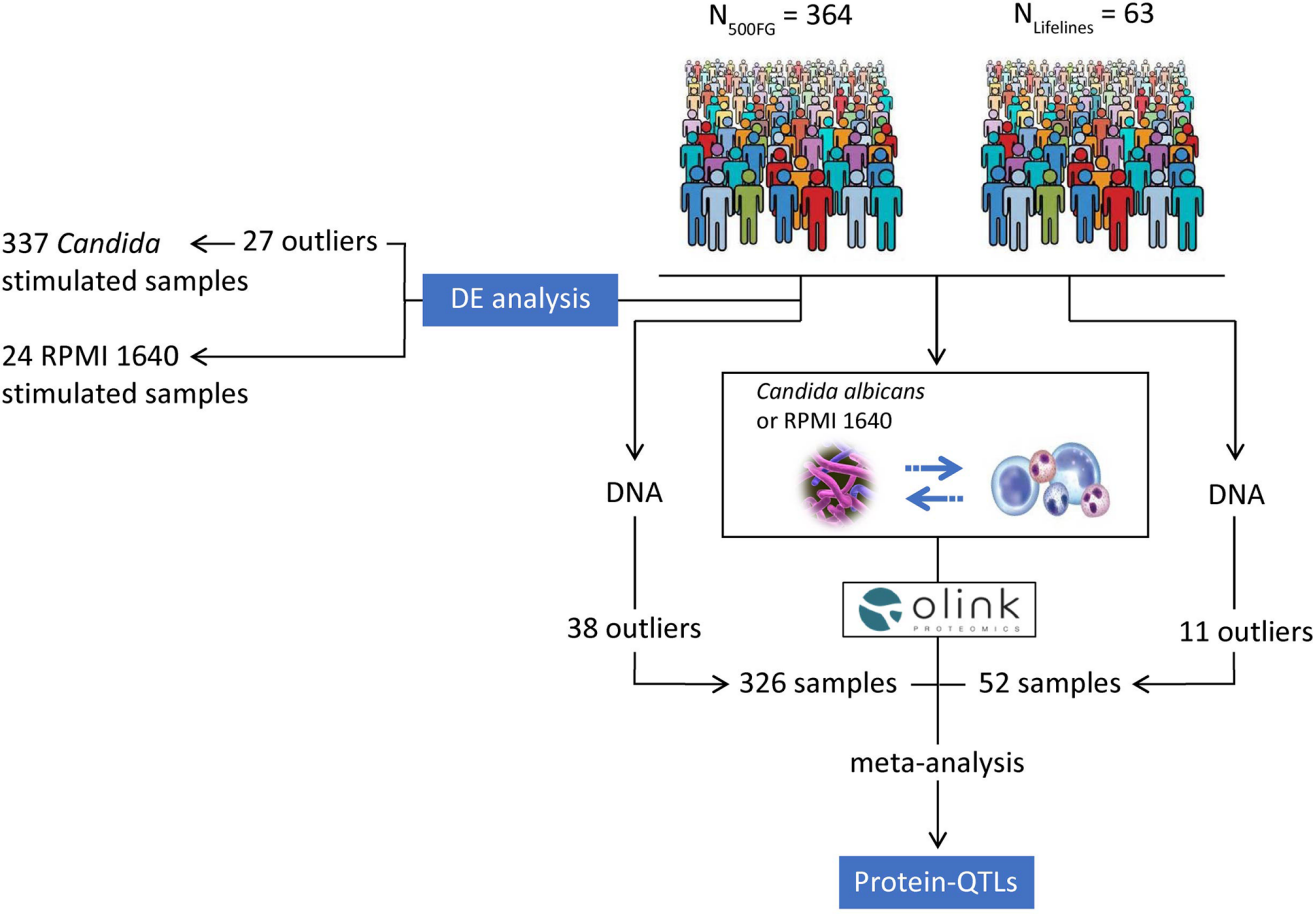
Overview of our study. We profiled the proteins released from *C. albicans*-stimulated PBMCs isolated from healthy individuals belonging to the 500FG (n = 364) and Lifelines Deep cohort (n = 63). We first compared the expression of proteins from *Candida*-stimulated samples (n = 337) with RPMI 1640-stimulated samples (n = 24) from the 500FG cohort to identify proteins that were significantly differentially expressed between the two conditions. Before DE protein analysis, we removed outliers that failed QC from OLINK proteomics (n = 26 samples) and one additional sample which had no age and sex information, resulting in 337 samples for DE analysis. In addition, we studied the effect of host genetics on the regulation of inflammatory responses by performing a meta-analysis of pQTLs mapped in the 500FG and Lifelines Deep cohorts. For pQTL mapping, we used the proteomic data measured in the 500FG (n = 364) and Lifelines Deep cohort (n = 63). We also obtained the imputed genotypes of the studied individuals. After removing outliers based on the quality control of the genotypes and proteomic data, the total number of samples used for pQTL mapping was 326 and 52 samples in the 500FG and Lifelines Deep cohort respectively. After quality control, we correlated the protein measurements with genome-wide SNP data in each cohort and performed meta-analysis using the summary statistics of pQTLs mapped in the two cohorts to identify genetic variants that influence the levels of inflammatory proteins. Stimulations of PBMCs were performed for 24 hours using *C. albicans* yeast and RPMI 1640 medium as control.

The aims of the present study were to identify the abundance of differentially regulated inflammatory proteins in peripheral blood mononuclear cells (PBMCs) stimulated with *C. albicans* yeast, and to investigate whether genetic variants influence inflammatory proteins (pQTLs) in the context of *Candida* infection (**Figure 1**). For this, we profiled 91 inflammatory proteins in the PBMCs isolated from two independent population-based cohorts, the 500 Functional Genomics (500FG) cohort of the Human Functional Genomics Project (HFGP, http://www.humanfunction-algenomics.org), and Lifelines Deep cohort (https://www.lifelines.nl/). We observed that the majority of inflammatory proteins were significantly increased in *Candida*-stimulated PBMCs compared to control PBMCs stimulated with RPMI 1640 medium. In addition, association of genetic variation with protein abundance identified 5 independent novel, genome-wide significant protein quantitative trait loci (pQTLs, *P* < 5 x 10^-8^). Lastly, we investigated whether pQTLs determine patient survival in candidaemia, and identified a locus on chromosome 3 that may determine survival through modulating urokinase plasminogen activator, also known as urokinase (uPA).

## Materials and Methods

### Study Populations

#### Population-Based Cohorts

To understand the variation in circulating inflammatory proteins in humans in response to *C. albicans in vitro*, we used two independent population-based cohorts, the 500FG, and the Lifelines Deep cohorts.

##### 500FG

The 500FG cohort is composed of healthy individuals of Western European ancestry from the Human Functional Genomics Project (HFGP, see www.humanfunctionalgenomics.org). The 500FG cohort is composed of 533 well-characterized healthy individuals, 237 males and 296 females, all between 18 and 75 years old.

*Demographic Data Collection in 500FG*. Volunteers, after donating blood in the hospital, received an extensive online questionnaire about lifestyle, diet, and disease history. Based on the results of this questionnaire, 45 volunteers were excluded for various reasons, e.g., they were under medication, non-European ancestry, or had a chronic disease. These exclusion criteria were taken to minimize false positive effects on the protein production capacity *in vitro*.

##### Lifelines Deep Cohort

Participants of the northern Netherlands population cohort LifeLines Deep (13) are part of the Lifelines cohort in the Netherlands (www.lifelines.nl/researcher/cohort-and-biobank). This unique Lifelines cohort is consisted of 1539 participants aged 18 years and older, of which multiple data levels are available for follow-up research.

***Candidaemia Cohort.*** Enrollment of candidaemia patients occurred between January 2003 and January 2009. The demographic and clinical characteristics of the candidaemia cohort have been described previously (14). For this study, a subgroup of patients consisted of 171 individuals, which were recruited at the Duke University Hospital (DUMC), were followed prospectively for up to 3 years and survival outcome was recorded for these individuals. Of these individuals, 115 were identified as non-survivors and 56 as survivors. These candidaemia patients had European ancestry and must have had at least one positive blood culture for a *Candida* species, with the majority of them infected by *C. albicans.*

#### Genotyping, Quality Control, and Imputation of the Study Populations

##### 500FG Cohort

Genotyping, quality control (QC) and imputation of the 500FG cohort were previously described (9). Briefly, DNA obtained and was genotyped using the commercially available Illumina HumanOmniExpressExome-8 v1.0 SNP chip. Upon QC per sample and SNP, genotypes were imputed using the Genome of the Netherlands (GoNL) as a reference panel (15). Before QTL mapping, we performed standard quality control per sample and SNP using the imputed genotypes of 482 samples. Upon QC per sample (**Supplementary Figure 1**), four samples were removed as duplicated samples and 18 samples due to high relatedness (PI_HAT > 0.2). As a measure of relatedness, we used the estimates of PI_HAT (proportion identity-by-decent) as calculated using plink v1.90b (16). One additional sample was removed due to reduced heterozygosity rate (3 standard deviations below the mean of heterozygosity rate). Excess heterozygosity rate may indicate sample contamination and reduced heterozygosity may indicate inbreeding. Lastly, 11 samples were removed as ethnic outliers based on the multidimensional scaling analysis (performed in PLINK on the N X N matrix of genome-wide IBS pairwise distance) (MDS) using the first two principal components (PC1 and PC2) (**Supplementary Figures 2A, B**). Ethnic outliers are defined as the samples which showed a value of PC1 and/or PC2 three standard deviations above or below the mean of PC1 and PC2. In total, we identified 30 genetic outliers of which 12 had protein measurements. Finally, we removed genetic variants with MAF < 5%, resulting in a total number of 5,464,689 variants that were used for follow-up pQTL mapping.

##### Lifelines Deep Cohort

Genotyping, quality contol and genotype imputation of the Lifelines Deep cohort have been published elsewhere (17). Briefly, DNA was isolated and genotyped using both the HumanCytoSNP-12 BeadChip and the ImmunoChip platforms (Illumina, San Diego, CA, USA). First, SNP quality control was applied independently for both platforms, and next the genotypes generated from both platforms were merged into one dataset. After merging and quality control, genotypes were imputed using the GoNL reference panel (15). For this study, we used 63 *Candida*-stimulated PBMC samples from which we measured proteins from the supernatant using OLINK proteomics as described below. Before QTL mapping, we performed QC per sample and SNP using the imputed genotypes as described for the 500FG cohort (**Supplementary Figure 1**). In total, we removed 11 outliers due to high relatedness, resulting in a total number of 52 samples for follow-up QTL mapping. Finally, we removed genetic variants with MAF < 5%, resulting in a total number of 5,464,689 variants that were used for pQTL mapping.

##### Candidaemia Cohort

Genotyping, QC and imputation of the candidaemia cohort were previously described (11). Isolated DNA obtained from candidaemia patients was genotyped using the commercially available SNP chips, HumanCoreExome-12 v1.0 and HumanCoreExome-24 v1.0 BeadChip from Illumina (https://www.illumina.com). Genotypes were imputed using the Michigan imputation server with the human reference consortium (HRC) as a reference panel to increase the number of SNPs to ~5 millions (18). After imputation, we performed standard QC per sample and SNP using our survival cohort consisted of 171 candidaemia patients. The survival cohort consisted of 115 non-survivors and 56 survivors. After excluding genetic variants with a MAF < 0.01, we applied QC per sample and removed 3 non-survivors due to high relatedness (PI_HAT ~ 0.99). We also removed 3 non-survivors and 1 survivor due to excess/reduced heterozygosity that show a value of heterozygosity rate 3 standard deviations above or below the mean of heterozygosity rate respectively. In addition, we identified 5 non-survivors and 3 survivors as ethnic outliers based on MDS of candidaemia cases using the first two principal components (**Supplementary Figures 3A, B**). After QC per SNP and sample, we tested for association with survival of ~5 million SNPs with MAF > 0.01 using 104 non-survivors and 52 survivors as described below.

### PBMC Collection and *Candida* Stimulation Experiments

Venous blood from the cubital vein of healthy volunteers was drawn in 10 ml EDTA Monoject tubes (Medtronic, Dublin) after obtaining written informed consent. PBMC isolation was performed as previously described (9). In short, the PBMC fraction was obtained using density centrifugation of EDTA blood diluted 1:1 in pyrogen-free saline over Ficoll-Paque (Pharmacia Biotech, Uppsala). Cells were then washed twice in saline and suspended in RPMI 1640 medium supplemented with gentamycin (10 mg/mL), L-glutamine (10 mM) and pyruvate (10 mM). PBMCs were counted in a Coulter counter (Beckman Coulter, Pasadena) and re-suspended in a concentration of 5 x 10^6^ cells/mL. A total of 5 x 10^5^ PBMCs were added in 100 μL to round-bottom 96-well plates (Greiner) and incubated with 100 μL of stimulus (heat-killed *Candida albicans* yeast of strain ATCC MYA-3573, UC 820, 1 x 10^6^/mL) or RPMI 1640 medium. After 24 hours, the supernatants were collected and stored at −20°C until assayed using the proximity extension assay (PEA) that is being used from Olink Proteomics (https://www.olink.com/).

### Fungal Growth Conditions

*C. albicans* ATCC MYA-3573 (UC 820) was grown overnight to generate yeast cells in Sabouraud dextrose broth at 29°C, with shaking. Cells were harvested by centrifugation, washed twice with PBS, and resuspended in culture medium (RPMI 1640 Dutch modification). *C. albicans* yeast was heat killed for 30 min at 95°C.

### RNA Sequencing and Differential Expression Analysis Using the GoNL Cohort

All bulk RNA-seq data from PBMCs was previously generated (10) in 70 individuals from the GoNL cohort (15) This data was generated from PBMCs that were stimulated for 24 h with *Candida* or remained unstimulated (RPMI 1640 control condition). Principal components analysis (PCA) of the gene expression data generated from the *Candida-* and RPMI 1640-stimulated PBMCs showed that two samples were possibly mislabelled, which were subsequently removed before differential expression (DE) analysis (**Supplementary Figure 4**). The differentially expressed genes upon stimulation were identified through DESeq2 package from Bioconductor using 72 *Candida*-stimulated- and 75 RPMI 1640-stimulated samples (19). The statistically significant threshold (FDR < 0.05) was applied.

### Proteomic Profiling of Circulating Inflammatory Proteins

Supernatant samples of PBMCs stimulated with *C. albicans* yeast and with RPMI 1640 control medium were analyzed using the proximity extension assay (PEA) that is being used from Olink Proteomics (20). We selected the Olink^®^ inflammatory panel (Olink Bioscience AB, Uppsala, Sweden). This panel includes 92 inflammatory proteins. To note, due to technical issues identified in the brain-derived neurotrophic factor (BDNF) assay from the Olink proteomics, we were unable to report any data for this analyte, and thus, we reported in this study only 91 inflammatory proteins (**Table S1**). Olink proteomics delivers the protein measurements as Normalized Protein Expression (NPX) values, which are Cq values normalized by the subtraction of values for extension control, as well as inter-plate control. The scale is shifted using a correction factor (normal background noise) and reported in the Log_2_ scale. We performed normality test on both raw and log-transformed data using Shapiro-Wilk normality test, and a P > 0.05 was used as a threshold for normal distribution. The majority of proteins followed a non-Gaussian distribution. The distribution of all protein measurements generated from *Candida*-stimulated PBMCs from 500FG and Lifelines Deep cohort are shown in **Supplementary Figures 5A, B** respectively. In addition to QC for protein distribution, Olink Proteomics performed QC per sample during which samples that deviate less than 0.3 NPX from the median pass the quality control.

### Clustering Analysis

To assess the correlation structure between pairs of inflammatory proteins measured in at least 85% of samples from *Candida*-stimulated PBMCs isolated from the 500FG cohort, unsupervised clustering was performed using Spearman’s correlation as the measure of similarity. All statistical analyses were performed in R version 3.2.2.

### Differential Expression Protein Analysis

To test which proteins are differentially expressed in *Candida*-stimulated PBMC samples (n = 364) *versus* RPMI 1640-stimulated PBMC samples (n = 24) in the 500FG cohort, we performed a DE analysis using a linear model with age and sex as covariates. Twenty-six out of 364 *Candida*-stimulated PBMC samples did not pass QC per sample performed by OLINK proteomics as described above (**Figure 1**). All samples stimulated with RPMI 1640 control medium passed quality control. After removing outliers, 337 *Candida*-stimulated samples (one sample out of the 338 samples was removed due to missing phenotype information) and 24 RPMI 1640-stimulated samples used for DE analysis. The total number of proteins that were measured in at least 85% of all samples and tested for DE analysis was 36 proteins. For DE analysis, we used the R package Limma adjusted to protein data, which is originally being used for the analysis of gene expression data (21). Limma uses an empirical Bayes method to moderate the standard errors of the estimated log-fold changes. Proteins with an FDR < 0.05 and a logarithm of fold change (logFC) > 2 compared to RPMI 1640-stimulated samples were considered statistically significant. Normalized protein measurements (NPX values) for all samples used in the DE analysis are provided in **Table S2**.

### Protein QTL Mapping

We performed a meta-analysis of *Candida*-induced pQTLs by using the summary statistics of the pQTL datasets that were mapped in the two independent population-based cohorts, the 500FG and Lifelines Deep cohort. Lack of protein measurements for all available individuals restricted us to select 364 individuals from 500FG and 63 samples from Lifelines Deep cohort for whom both genotype and protein data were available (**Figure 1** and **Supplementary Figure 1**). In addition to the outliers that did not pass the QC from Olink Proteomics, we removed genetic outliers before QTL mapping as described above. In total, we removed 14 genetic outliers from the 500FG cohort and 11 genetic outliers from the Lifelines Deep cohort, resulting in 326 samples from 500FG cohort and 52 samples from the Lifelines Deep cohort (**Supplementary Figure 1**). To correct the protein distributions for QTL mapping, we used a linear model adjusting for age and sex. NPX values of protein levels were used, and the correlation between protein abundance and genotype was tested independently for the two cohorts using the R-package Matrix-eQTL (22).

Next, we performed meta-analysis using the summary statistics of pQTLs that influence proteins that were measured in at least 85% of the samples in both cohorts. HGF protein was one of the proteins that was not measured sufficiently in the 500FG cohort in contrast to the Lifelines Deep cohort (**Table S1**), and, hence, the number of proteins that used for meta-analysis was 35 proteins. To jointly test the effect of genetic variation in protein levels measured in both 500FG and Lifelines cohort, we used METAL (http://www.sph.umich.edu/csg/abecasis/metal/) using 35 proteins from 326 samples of the 500FG cohort and 52 samples of the Lifelines Deep cohort. Finally, we removed pQTLs from meta-analysis with opposite direction and those that show significant heterogeneity (*P* < 0.05) between the two cohorts (23). We considered *P* < 5 x 10^-8^ to be the threshold for genome-wide significant pQTLs.

### Genotype-Dependent Gene Expression Analysis at rs10114707 Locus

Both genotype and gene expression data upon *Candida* stimulation were available from 62 individuals of the GoNL cohort (15). The stimulation experiments and the RNA sequencing analysis of this dataset has been previously described (10). There were 32, 22 and 8 individuals carrying AA, GA and GG genotypes respectively at rs10114707 locus. We combined GA and GG in one group and performed DE analysis between two genotype groups (GA+GG *versus* AA) using the expression data of *cis*-genes (n = 30) extracted from the 5 GWAS loci (in a window of 1MB around each GWAS locus), which were identified in the meta-analysis of protein-QTLs. For DE analysis using gene counts, we used the DESeq2 package (19). Summary statistics of the DE analysis is shown in **Table S3**.

### Pleiotropy

To test all reported genome-wide significant pQTLs for pleiotropy, we initially inspected all genome-wide significant *trans*-pQTLs whether they show association with multiple distinct proteins. To correct for the multiple testing burden for all genome-wide pQTLs, we next inspected all genome-wide significant *trans*-pQTLs that had at least two associations with distinct proteins at *P* < 0.05/(5*35) = 0.0003. This cut-off represents a conservative approach to the multiple testing burden for all identified, independent GWAS SNPs (n = 5) with all protein traits (n = 35). The resulting association matrix was then visualized as a heatmap based on the negative log2 of the P values of association.

### Within Case/Control Association Analysis for Candidaemia Survival

To identify genetic variants that are associated with survival, we ran a within-case genome-wide association study using our survival cohort consisted of 171 candidaemia patients, of which 115 were identified as non-survivors and 56 as survivors. After quality control per SNP and sample, we tested for association with survival of ~5 million SNPs using 104 non-survivors and 52 survivors. Age and sex were included as covariates in an additive model using a frequentist association test from SNPTEST v.2.5.4-beta3 (24). The quantile-quantile (QQ) plot that shows the distribution of the observed negative log_10_ P values for the whole-genome SNPs against the theoretical distribution of expected negative log_10_ P values indicated no or little evidence of population stratification (**Supplementary Figure 6**). The genomic inflation factor based on median was equal to λ = 1.03.

## Results

### Overview of Inflammatory Protein Profiles in PBMCs From Healthy Individuals in Response to *C. albicans*

To systematically study the inflammatory responses in the context of *C. albicans* infection, we first obtained PBMCs from 364 individuals of European origin from the 500FG cohort. We next measured the abundance of 91 inflammatory proteins in the supernatant of PBMCs in response to *C. albicans* yeast stimulation (**Figure 1**). Of these proteins, 36 were detected in at least 85% of the samples (**Table S1**), and all proteins showed significant inter-individual differences to *C. albicans* stimulation compared to RPMI 1640 medium stimulation (**Supplementary Figure 7**). After removing samples that failed the QC (n = 24) and a sample with no age and sex information, we compared the abundance of the 36 proteins between stimulated (n = 337) and un-stimulated (using RPMI 1640 medium as control, n = 24) conditions by performing DE protein analysis using a linear model with age and sex as covariates. We identified significant up-regulation of 25 proteins out of 36 measured in at least 85% of PBMC samples that showed logFC > 2 compared to RPMI 1640-stimulated samples (adjusted *P* value < 0.05) (**Supplementary Figure 8A** and **Table S4**). Next, we compared the correlation structure among the inflammatory proteins released from PBMCs by performing unsupervised clustering of the protein responses. Clustering revealed that the majority of proteins showed a strong positive correlation (*P* < 0.01) (**Supplementary Figure 8B**, **Table S5**), with the exception of IL-8 and MCP-1, indicating the coordinated inflammatory protein response upon *Candida* stimulation.

### Identifying Genetic Variation Affecting Inflammatory Proteins in Response to *C. albicans*

**Figure 2:**
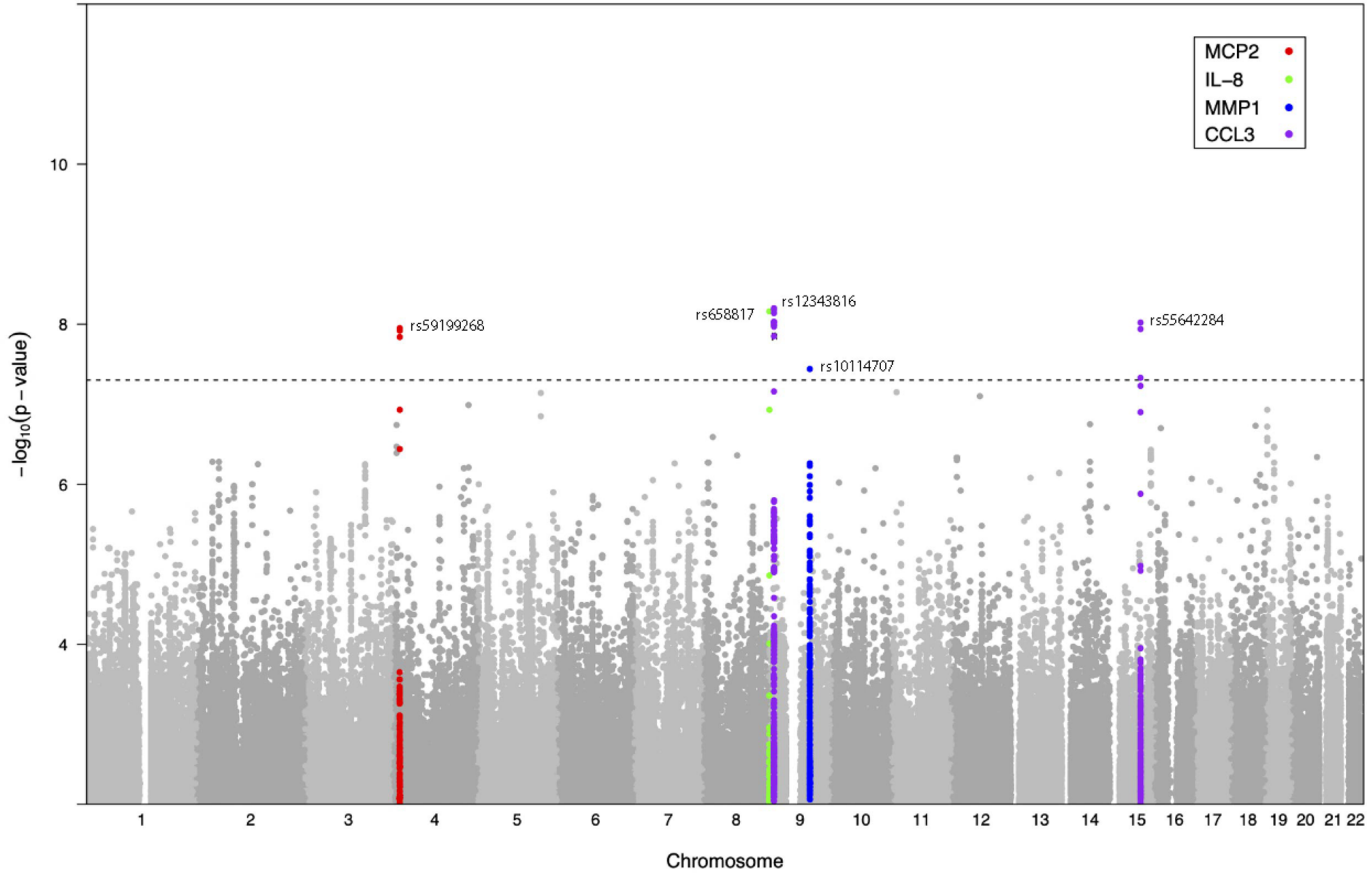
Genome-wide protein-QTL mapping identified five *C. albicans* yeast-induced pQTLs. Manhattan plot showing the genome-wide QTL mapping results for *C. albicans*-induced MCP-2 (red), IL-8 (green), MMP-1 (blue), and CCL3 levels (purple). The y-axis represents the negative log_10_ P values of pQTLs. Their chromosomal positions are shown on the x axis. The horizontal dashed line represents the genome-wide significance threshold for association (*P* < 5 x 10^-8^).

Next, we investigated whether host genetic variation affects the inter-individual differences in inflammatory responses to *C. albicans* stimulation. For this, we used the genome-wide SNP genotype data and protein measurements of *Candida*-stimulated PBMCs isolated from two independent population-based cohorts consisting of 364 individuals from 500FG cohort and 63 individuals from Lifelines Deep cohort. Upon quality control (see Materials and Methods), we obtained 326 samples from 500FG cohort and 52 samples from the Lifelines Deep cohort with a total of 35 inflammatory proteins that were measured in at least 85% of the samples in both cohorts. For pQTL mapping, we selected SNPs that showed a minor allele frequency (MAF) >= 5% and passed other quality filters (see Materials and Methods). Using the protein and genotype data, we mapped pQTLs using each cohort independently and performed meta-analysis using the summary statistics of pQTLs identified in each cohort (n = 378). Meta-analysis revealed five independent *trans*-pQTLs that reached genome-wide significance level (*P* < 5 x 10^-8^, *P* for heterogeneity > 0.05) (**Table 1** and **Figure 2**). These include one independent pQTL for IL-8 (chromosome 9), one for MCP-2 (chromosome 4), one for MMP-1 (chromosome 9) and two for CCL3 (chromosome 9 and 15).

**Table 1:** Genome-wide significant pQTL loci that were identified in the meta-analysis of pQTLs identified in the 500FG and Lifelines Deep cohort.

Chr	SNP	BP	Allele1	Allele2	MAF	Z score	P-value	Protein	*cis*-gene(s)
4	rs59199268	16118500	A	G	0.08	5.71	1.11x10^-8^	MCP-2	*TAPT1*^a,b^, *CD38*^b^, *FAM200B^b^*, *FGFBP2^b^*, *LDB2^b^*, *TAPT1-AS1^b^*, *FGFBP1^b^*, *BST1^b^*, *FBXL5^b^*
9	rs12343816	11477574	T	C	0.18	5.81	6.37x10^-9^	CCL3	*HRASLS2*^c^, *NRP1*^c^, *TMEM38A*^c^
9	rs10114707	92309687	G	A	0.27	5.51	3.64x10^-8^	MMP-1	*GADD45G*^b^, *UNQ6494*^b^, *CKS2^b^*, *SECISBP2^b^*, *SEMA4D^b^*
9	rs658817	1439567	T	G	0.05	5.79	6.87x10^-9^	IL-8	*RN5S279*^d^, *DMRT2*^d^
15	rs55642284	69922759	A	G	0.16	5.74	9.62x10^-9^	CCL3	*GLCE^b^, LINC00593^b^, PAQR5^b^, KIF23^b^, TLE3^b^, RPLP1^b^, RP11-279F6.3*^d^, *LOC145837*^d^

a SNP or proxy SNP (LD > 0.8) shows an expression-QTL (eQTL) effect in blood (eQTLGen Consortium).

b Gene is differentially expressed upon C. albicans stimulation at 24 hours.

c SNP or proxy SNP (LD > 0.8) shows an expression-QTL (eQTL) effect in peripheral blood monocytes.

d Closest annotated genes to pQTL.

MAF, Minor allele frequency calculated in the combined cohort of 500FG and Lifelines Deep cohort; Chr, chromosome; BP, Position in base-pairs; Allele1, Minor allele; Allele2, Major allele.

### Prioritization of *cis*-Genes at pQTLs That Affect Protein Abundance In Circulation in Response to *C. albicans*

**Figure 3:**
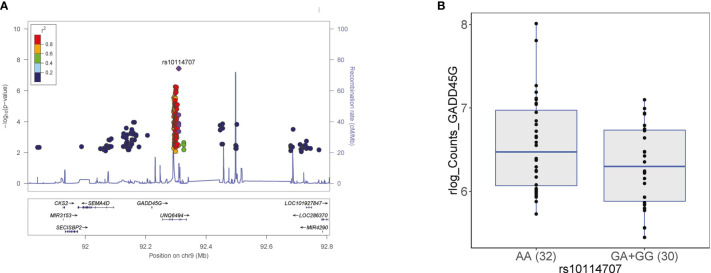
**(A)** Regional association plot at rs10114707 (purple diamond) that influences MMP-1, which is in close proximity to *GADD45G* gene at chromosome 9. Each dot represents a SNP, which shows a meta-analysis *P* value < 0.05 and the same direction in the 500FG and Lifelines Deep cohort. The linkage disequilibrium (LD) of neighboring SNPs with the top SNP is color-coded. The y-axis represents negative logarithm of *P* values of SNPs. The x-axis shows chromosomal positions on Genome build GRCh37 (hg19). **(B)** Genotype-stratified gene expression levels of *GADD45G* at rs10114707 locus. Individuals carrying the minor allele G at this locus showed a decrease in *GADD45G* expression. We presented the regularized-logarithm (rlog; transformed data on the log2 scale) of the counts of *GADD45G* gene from the two-genotype groups (GA+GG *versus* AA).

In these five independent loci, we prioritized potential causal genes following two approaches. First, we tested whether genes that are located within 1MB window around the genome-wide significant SNPs were differentially expressed in response to *C. abicans* yeast stimulation after 24 hours in PBMCs using our previously published *Candida*-induced transcriptomic dataset generated from the Dutch population-based cohort, GoNL (**Table S6**) (10). Secondly, we made use of the largest *cis*- and *trans*-expression quantitative trait locus (eQTL) study in blood from a total of 31,684 individuals through the eQTLGen Consortium (25) to test if the genome-wide SNPs affect gene expression in whole blood (**Table 1**) or in human monocytes (26). Our strategy prioritized several interesting genes including *CD38* and *GADD45G*. Briefly, *CD38* gene is prioritized from the rs591999268 locus at chromosome 4 based on its significant differential expression upon 24-hour *Candida* stimulation (adjusted *P* 9.75 x 10^-50^, log2 of fold change = 3.25). This finding suggests that CD38 may affect protein levels upon C. *albicans* stimulation. CD38 is a glycoprotein widely expressed in cells from the immune system, and animal studies indicate that it confers protection against infection by several bacterial and parasitic pathogens (27), suggesting its role on regulation of inflammatory protein response. As a second example, growth arrest and DNA damage inducible gamma (*GADD45G)* gene from the rs10114707 locus at chromosome 9 was prioritized based on two levels of evidence. Firstly, *GADD45G* is significantly differentially expressed in response to *C. albicans* at 24 hours (adjusted *P* 4.61 x 10^-97^, log2 of fold change = 2.63). Secondly, we tested if *GADD45G* gene is differentially expressed among individuals carrying different genotypes (AA, GA and GG) at rs10114707, which could explain how SNP rs1011477 can affect the levels of inflammatory proteins. For this, we stratified individuals from the GoNL cohort based on their genotype at rs10114707 locus and tested whether cis-genes in a window of 1MB around each GWAS locus is differentially expressed. We observed that individuals heterozygous or homozygous for the minor allele G at rs10114707 tend to show a decrease in *GADD45G* expression compared to individuals homozygous for allele A (**Figure 3** and **Table S3**). Such stimulation specific expression QTLs will be helpful to identify potential regulators of inflammatory protein response.

### Pleiotropy of Loci Affecting Protein Levels

**Figure 4:**
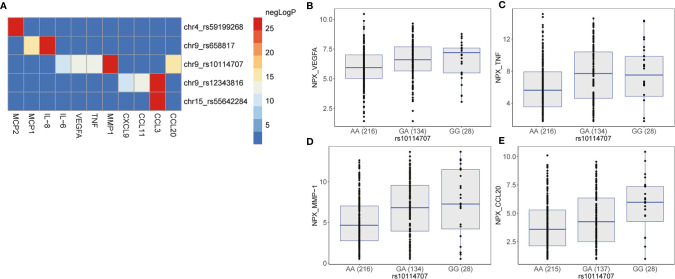
Pleiotropy between genome-wide significant pQTLs and inflammatory proteins measured using the proximity extension assay (PEA) by Olink proteomics. Heatmap **(A)** shows the negative log_2_(*P*) of all possible associations of the genome-wide significant pQTLs and the measured inflammatory proteins that show the same allelic direction between 500FG and Lifelines Deep cohort at a P value of 0.0003, and P_heterogeneity_ ≥ 0.05. Boxplots showing the expression levels (as NPX values) of **(B)** VEGFA, **(C)** TNF, **(D)** MMP-1, and **(E)** CCL20 when individuals from the 500FG and Lifelines Deep cohorts stratified by genotype at the genome-wide locus, rs10114707.

It is possible that the same genetic locus may regulate multiple proteins (pleiotropy). To test the presence of such pleiotropic effects, we extracted all possible associations of genome-wide significant pQTLs with the 35 proteins in the same allelic direction between the two cohorts. We observed that all genome-wide significant pQTLs influence multiple distinct proteins (**Figure 4A** and **Table S7**), indicating the pleiotropic effects of the pQTLs on the proteins. To account for multiple testing, we inspected all genome-wide significant *trans*-pQTLs that had at least two associations with distinct proteins at *P* = 0.05/(5*35) = 0.0003. This cut-off represents a conservative approach to correct for multiple testing for all identified GWAS SNPs (n = 5) with all protein traits (n = 35). SNP rs59199268 at chromosome 4 and rs55642284 at chromosome 15 showed only a single association with MCP2 and CCL3, respectively. We observed three loci on chromosome 9 at genetic variants rs10114707, rs12343816 and rs658817 that influence four, two and another two proteins respectively at *P* < 0.0003 in the same allelic direction (**Table S7**). These pleiotropic effects suggest that these proteins are co-regulated in inflammation induced by *C. albicans*. For instance, individuals carrying the minor allele G at rs10114707 seem to upregulate the levels of VEGFA, TNF, MMP-1, and CCL20 (**Figures 4B–E**). We prioritized *GADD45G* as a potential causal gene at this locus as discussed above. GADD45 proteins are known to be induced by a number of cytokines and by bacterial lipopolysaccharide, have an essential role in the differentiation of myeloid cells as well as in the function of granulocytes and macrophages (28, 29). Given this important immune-regulatory function it is plausible that GADD45G could be one of the core regulators of multiple inflammatory proteins. However, more studies are needed to explain the specific mechanism by which these pleiotropic effects occur.

### Contribution of pQTLs That Influence Circulatory Inflammatory Proteins to Survival to Candidaemia

**Figure 5:**
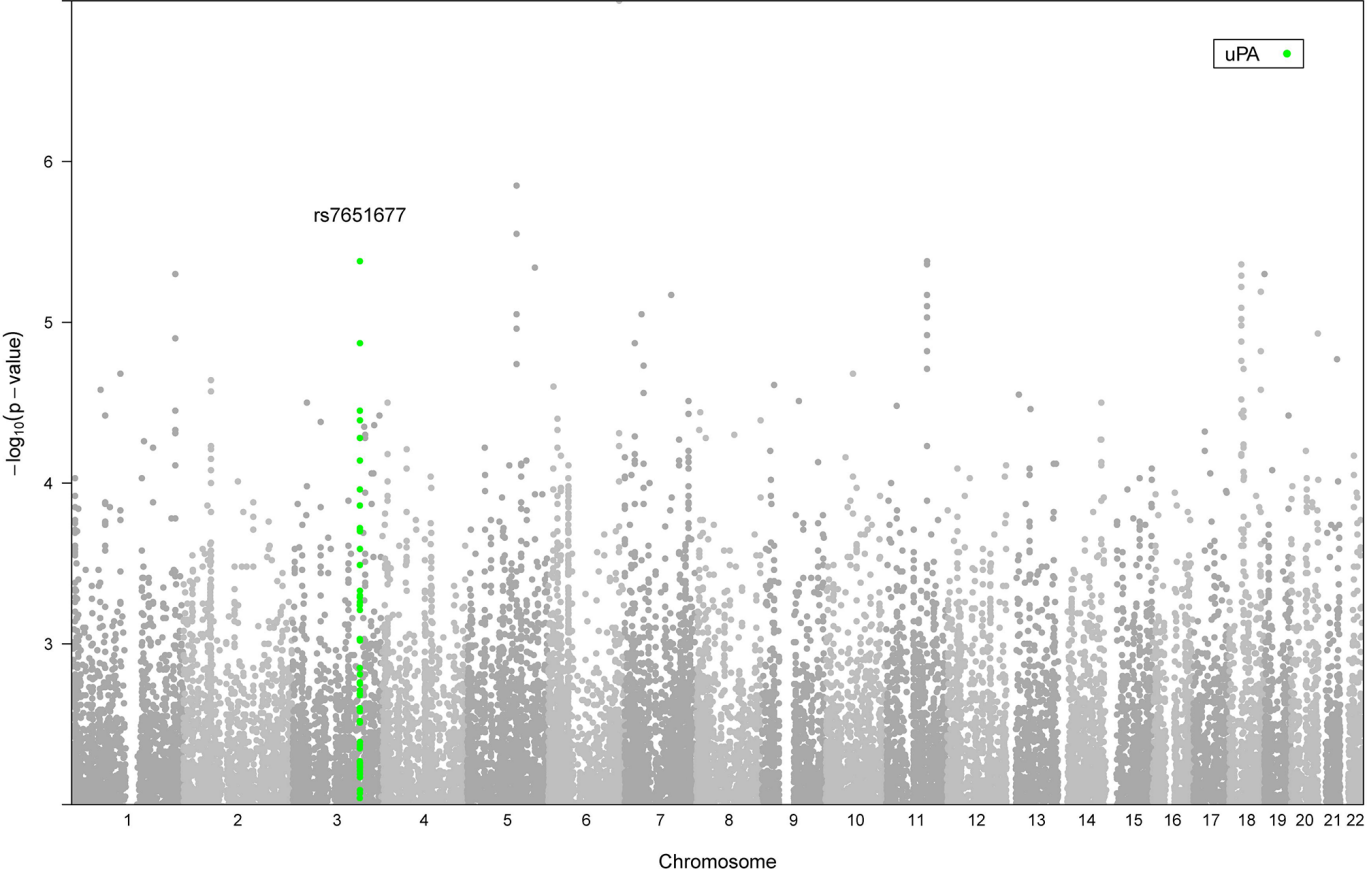
Manhattan plot showing the genome-wide P values of association with survival. The y axis represents the negative log10 P values and their chromosomal positions are shown on the x axis. The locus marked at SNP rs7651677 on chromosome 3 that influences uPA (*P* 8.29 x 10^-6^, z score 4.46) and shows a strong association with survival (*P* 3.52 x 10^-5^ and OR 3) is highlighted with green.

Next, we aimed to investigate whether genetic variation that influences inflammatory proteins (pQTLs) in blood circulation may determine patient survival to candidaemia. To identify genetic variants that are associated with survival in candidaemia patients, we performed a within-case GWAS analysis using 156 individuals, of which 104 were identified as non-survivors and 52 as survivors. After QC per SNP and sample, we tested 5450489 variants (MAF > 1%) for association with survival using an additive model with age and gender as covariates. No genome-wide significant associations were observed (*P* < 5 x 10^-8^), which can be explained due to the small number of patients with reported survival outcome (**Figure 5**). To test whether genetic variants that are associated with survival influence inflammatory proteins in response to *Candida*, we extracted the within-case GWAS summary statistics of survival and overlaid this with our trans-pQTLs at a *P* threshold 1 x 10^-4^. A trans-pQTL, rs7651677, at chromosome 3 that influences uPA (*P* 8.29 x 10^-6^, z score 4.46), also known as urokinase, showed a significant association with survival (*P* 3.52 x 10^-5^ and OR 3), indicating that this locus may determine survival of candidaemia patients through modulating uPA. In this regard, it is interesting to note that the serum suPAR (soluble urokinase plasminogen activator receptor) levels are considered as a biomarker to assess the risk in sepsis (30).

## Discussion

The incidence of opportunistic invasive fungal infection has been increased over the last decades. This increase can be explained by the use of aggressive and intensive chemotherapeutic regimens, immunosuppressive therapy for autoimmune disorders, and transplantation that have led to a rise in the number of susceptible human hosts (31). The development of specific and mechanistically relevant plasma biomarkers in *Candida* infections are therefore important tools in identifying patients at high-risk and, thus, clinicians can provide the most beneficial prophylactic and/or treatment strategy. In this study, we performed a systematic proteomic analysis of plasma levels of inflammatory-related proteins in PBMC samples in response to *Candida* stimulation. To our knowledge, this is the first effort to date to evaluate a great number of inflammatory proteins and assess the role of host genetics in regulating inflammatory protein levels in *Candida* infection.

We observed an increased inter-individual variation in inflammatory responses in PBMCs stimulated with *C. albicans* compared to RPMI 1640 medium-stimulated samples (**Supplementary Figure 7**), suggesting that (non)-genetic factors influence cytokine production capacity in humans. Previous studies have shown that age, sex and seasonality are important factors that influence immune responses (32). By studying the most important pro-inflammatory cytokines produced by monocytes, Th1 and Th17 cells (IL-6, TNF-α, IL-1β, IL-17, IL-22 and IFN-γ), we showed that host genetics play a major role in inter-individual variability in cytokine responses (9). In this study, to explain the observed inter-individual variations in a broader spectrum of inflammatory responses, we investigated the contribution of genetic factors to inflammatory responses by performing a meta-analysis of pQTLs using two independent population-based cohorts, the 500FG and Lifelines Deep cohort. We identified 5 independent pQTLs with MAF > 5% that reached genome-wide significance level (*P* < 5 x 10^-8^) that influence IL-8, MCP-2, MMP-1, and CCL3. Similar to previous findings (9), all pQTLs that we find are *trans*-QTLs, and thus, influence inflammatory responses indirectly through regulatory loops. These findings not only help to identify specific genetic variants that affect cytokine levels but also provide insights into trans-regulatory mechanisms. For example, we found a SNP, rs10114707, near *GADD54G* gene that affects not only MMP-1 levels but also four other inflammatory protiens (VEGFA, TNF, IL-6 and CCL20, **Table S7**). *GADD45G* encodes a protein that belongs to the family of Gadd45 proteins that are important components of intracellular signaling networks that are activated by a number of cytokines and lipopolysaccharide, mediating intercellular communication in response to invading pathogens (28). GADD45G has been reported to interact with several proteins that are involved in DNA repair, cell cycle control, apoptosis and senescence (33–35). Therefore, we hypothesized that GADD45G could be a potential core gene that regulates a cytokine network upon *Candida* infection. Identifying such core genes that regulates cytokine networks will be critical to better understand inflammatory processes during infections.

Moreover, we investigated the contribution of trans-pQTLs identified in the meta-analysis to patient survival. Despite the small size of the patient cohort with known survival outcome, we observed that a single locus marked from SNP rs7651677 at chromosome 3 that influences uPA is strongly associated with patient survival (*P*_survival_ = 3.52 x 10^-5^, OR 3). Interestingly, the uPA has been previously reported to convert plasminogen to plasmin, which then activates matrix metalloproteinases (MMPs), such as MMP-9, degrading extracellular matrix structure. Previous mouse studies demonstrated that certain species of bacteria, such as Group A streptococcus, can recruit plasmin accumulation on the cellular surface, consequently degrading the extracellular matrix, and initiating bacterial invasion (36). Previous studies also showed that *C.albicans*, among other pathogenic bacteria, may use plasmin for its invasion by binding plasminogen as well as expressing a range of proteins that function as plasminogen receptors (37). Although inhibiting plasminogen activation by uPA is considered as a therapeutic target for invasive bacterial infection, more investigations are needed to establish the role of uPA and plasminogen in *Candida* infections.

There are also limitations to our study. First, the experimental setup of *ex vivo* PBMC stimulation for 24 hours with *C. albicans* yeast provides only the opportunity to study the interactions between immune cells, such as monocytes, T and B cells, missing the neutrophils and platelet fractions. Also, the use of heat-killed *C. albicans* does not fully represent the interactions between live *Candida* cells and PBMCs. Another limitation is that the time-dependent dynamic interactions are missing as PBMCs were stimulated for 24 hours. As such, other inflammatory responses, which are triggered by the late-stage proteins of *C. albicans* infections may be missed. In addition, it would be interesting to capture inflammatory responses upon stimulation with *C. albicans* hyphae, as the transition to hyphae contributes to *C. albicans* virulence, as well as other *C. albicans* strains or *Candida* species of different virulence, as some variability in terms of immunological responses may occur depending on the strain or species that is being used. In addition, although we validated the pQTLs in an independent cohort, because of relatively small sample size, we cannot exclude that some of the pQTLs were false positives. Also, it would be interesting to extend this study to populations other than Europeans as population-specific genetic factors may drive differences in inflammatory responses in response to *Candida*. In conclusion, we investigated in-depth the inflammatory proteins in response to *Candida* infection, and specifically the genetic variability that could modulate the expression of these proteins. Our genetic analysis suggested that *GADD45G* from rs10114707 locus that reached genome-wide significance could be a potential core gene that regulates a cytokine network upon *Candida* infection. We also investigated whether pQTLs determine patient survival in candidaemia patients and identified a single locus on chromosome 3 that affects uPA that is strongly associated with patient survival. This protein, which is known to interact with *C. albicans* may be a potential therapeutic target to improve patient outcome. Overall, this study provided an important groundwork to employ various -omics data in response to *Candida* infection using large population-based cohorts in the context of candidaemia where currently available patient cohorts are of limited size. This study can trigger critical future work to understand better the contribution of genetics to the inflammatory responses against *C. albicans* and how they shape survival in candidaemia patients.

## Data Availability Statement

The original contributions presented in the study are included in the article/**Supplementary Material**, further inquiries can be directed to the corresponding author.

## Ethics Statement

The studies involving human participants were reviewed and approved by the Ethical Committee of Radboud University Nijmegen, the Netherlands (no. 42561.091.12). The patients/participants provided their written informed consent to participate in this study.

## Author Contributions

VK and CW conceived the study and supervised the research. VM performed the analyses and drafted the manuscript. MJ, MDJ, AZ, and JF contributed to the acquisitions and collection of the samples and data. All authors contributed to the article and approved the submitted version.

## Funding

This work was supported by a Research Grant [2017] of the European Society of Clinical Microbiology and Infectious Diseases (ESCMID) and Hypatia tenure track grant to VK, European Research Council (ERC) Consolidator Grant [FP/2007-2013/ERC grant 2012-310372] and a Netherlands Organization for Scientific Research (NWO) Spinoza prize grant [NWO SPI 94-212] to MN, an ERC Advanced grant [FP/2007-2013/ERC grant 2012-322698] and an NWO Spinoza prize grant [NWO SPI 92-266] to CW, a European Union Seventh Framework Programme grant (EU FP7) TANDEM project [HEALTH-F3-2012-305279] to CW. YL was supported by a VENI grant (863.13.011) from the Netherlands Organization for Scientific Research (NWO).

## Conflict of Interest

The authors declare that the research was conducted in the absence of any commercial or financial relationships that could be construed as a potential conflict of interest.

## Publisher’s Note

All claims expressed in this article are solely those of the authors and do not necessarily represent those of their affiliated organizations, or those of the publisher, the editors and the reviewers. Any product that may be evaluated in this article, or claim that may be made by its manufacturer, is not guaranteed or endorsed by the publisher.

## Supplementary Material

Supplementary Figure 1 Flowchart of the quality control (QC) per sample, protein and SNP in the 500FG and Lifelines Deep cohort. The initial number of *Candida*-stimulated samples from the 500FG with protein measurements was 364 samples. Olink Proteomics performed QC per sample during which samples that deviate less than 0.3 NPX from the median pass the quality control. Twenty-six samples were failed QC from OLINK proteomics in the 500FG cohort. In addition, 12 samples were removed as genetic outliers. After removing outliers based on the proteomic and genotype data, the final number of samples used for pQTL mapping was 326 samples in the 500FG cohort. In the Lifelines Deep cohort, the initial number of *Candida*-stimulated samples with protein measurements was 63. All samples pass QC from OLINK proteomics, and 11 samples were removed as genetic outliers, resulting in a total number of 52 samples for pQTL mapping. Upon QC per protein, we used proteins that were measured in at least 85% of samples, resulting in a total number of 36 and 47 proteins in the 500FG and Lifelines Deep cohort respectively. In both cohorts, SNPs with MAF < 5% were removed before QTL mapping, resulting in approximately 5 M SNPs in each cohort. Finally, we used the summary statistics of pQTLs mapped in each cohort for meta-analysis. Proteins that were measured in at least 85% of all samples in both cohorts were used for meta-analysis, resulting in 35 proteins.

Supplementary Figure 2 Multidimensional scaling (MDS) analysis was performed on the 500FG cohort to identify ethnic outliers. **(A)** MDS plot before removing ethnic outliers and **(B)** after removing ethnic outliers. As genetic outliers were identified as those samples with PC1 and/or PC2 three standard deviations above or below the mean of PC1 and/or PC2.

Supplementary Figure 3 Multidimensional scaling (MDS) analysis was performed on candidaemia patients grouped in non-survivors (n = 115 individuals, green dots) and survivors (n = 56 individuals, yellow dots). **(A)** MDS plot highlighting the ethnic outliers. As ethnic outliers were identified as those samples with PC1 and/or PC2 three standard deviations above or below the mean of PC1 and/or PC2 (8 individuals, orange dots). **(B)** MDS plot after removing the ethnic outliers.

Supplementary Figure 4 Principal component analysis (PCA) for all genes measured in PBMC samples stimulated with *C. albicans* (green dots) *versus* RPMI 1640 medium-stimulated samples (yellow dots) in the GoNL cohort.

Supplementary Figure 5 Distribution plots of proteins measured in supernatant of *Candida*-stimulated PBMC samples from the **(A)** 500FG cohort and **(B)** Lifelines Deep cohort. The total number of proteins that were measured in at least 85% of samples was 36 proteins and 47 in the 500FG and Lifelines Deep cohort respectively. The total number of shared proteins between the two cohorts that were measured in at least 85% of the samples and used for meta-analysis was 35 proteins. Axis x represents the protein expression levels as NPX values (in Log_2_ scale).

Supplementary Figure 6 Quantile-Quantile (QQ) plot of association P-values for all SNPs on the genome-wide genotyping array (Black dots). The genetic inflation factor λ for all SNPs was 1.03, indicating no population stratification in the survival cohort of candidaemia patients.

Supplementary Figure 7 Inflammatory proteins showed significant inter-individual differences to *C. albicans* stimulation compared to RPMI 1640 medium control in the 500FG cohort.

Supplementary Figure 8 **(A)** Differential expression protein analysis in *Candida*-stimulated *versus* RPMI 1640 medium-stimulated PBMC samples from the 500FG cohort. Volcano plot of proteins showing significantly differentially expressed proteins with adjusted *P* < 0.05 (red). Benjamini-Hochberg method used to correct for multiple testing, and adjusted P values < 0.05 were considered significant. Age and sex used as covariates. **(B)** Unsupervised hierarchical clustering of inflammatory proteins that were measured in at least 85% of PBMC samples stimulated with *C. albicans* yeast. Spearman’s correlation was used as the measure of similarity. The blue color depicts the strong positive correlation whereas the red color indicates the strong negative correlation. Correlations between pairs of proteins that show a *P* value > 0.01 were crossed on the plot.

## Abbreviations

SNP, single-nucleotide polymorphism; PBMCs, peripheral blood mononuclear cells; GWAS, Genome-wide association study; pQTL, protein quantitative trait locus.
