# Supplementary figures and images for: Inflammatory Protein Profiles in Plasma of Candidaemia Patients and the Contribution of Host Genetics to Their Variability
Source: Front Immunol. 2021 Aug 26;12:662171. doi: 10.3389/fimmu.2021.662171 (PMC8428519; doi:10.3389/fimmu.2021.662171)

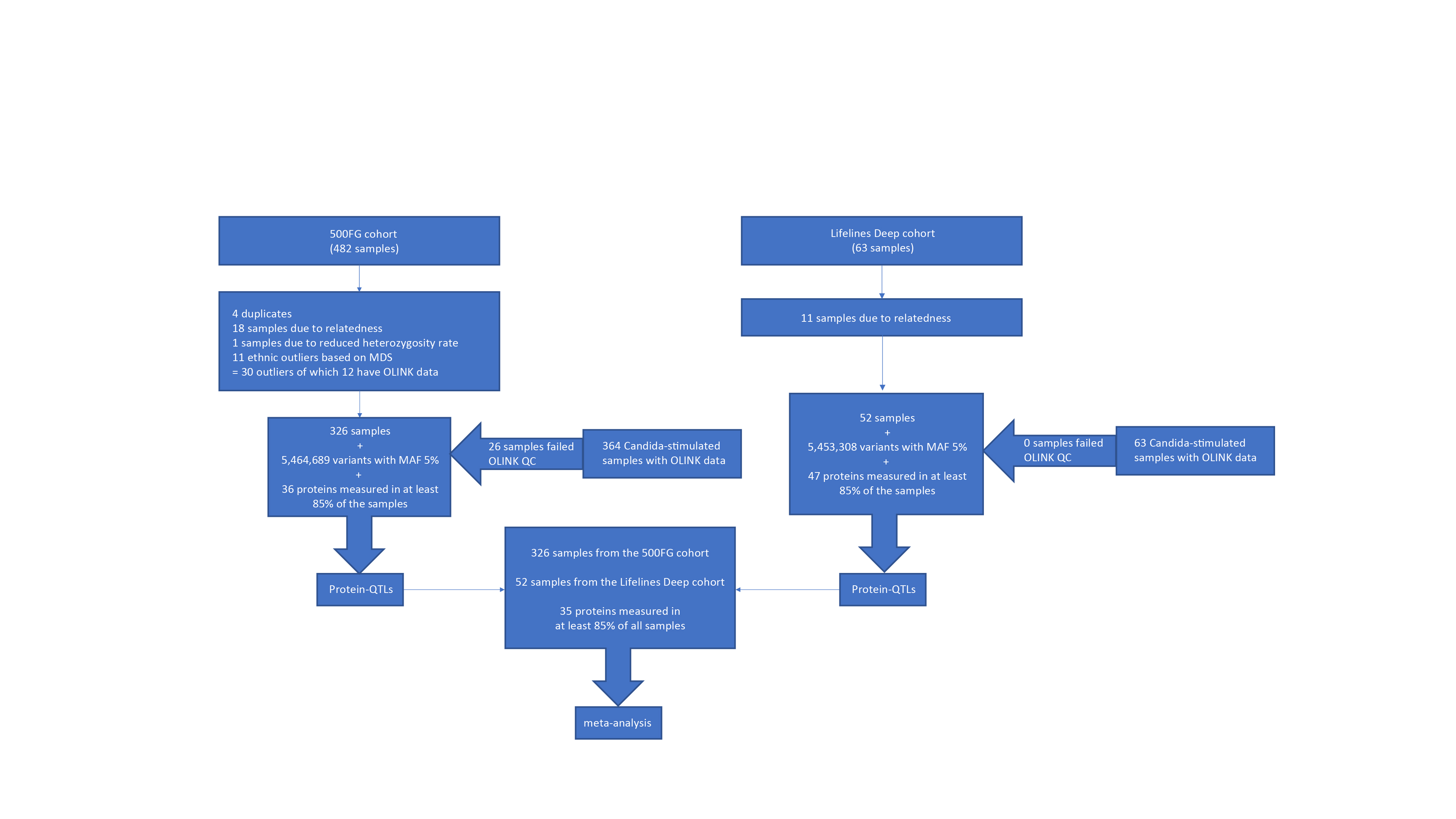

Supplement: Supplementary Figure 1 — Flowchart of the quality control (QC) per sample, protein and SNP in the 500FG and Lifelines Deep cohort. The initial number of Candida-stimulated samples from the 500FG with protein measurements was 364 samples. Olink Proteomics performed QC per sample during which samples that deviate less than 0.3 NPX from the median pass the quality control. Twenty-six samples were failed QC from OLINK proteomics in the 500FG cohort. In addition, 12 samples were removed as genetic outliers. After removing outliers based on the proteomic and genotype data, the final number of samples used for pQTL mapping was 326 samples in the 500FG cohort. In the Lifelines Deep cohort, the initial number of Candida-stimulated samples with protein measurements was 63. All samples pass QC from OLINK proteomics, and 11 samples were removed as genetic outliers, resulting in a total number of 52 samples for pQTL mapping. Upon QC per protein, we used proteins that were measured in at least 85% of samples, resulting in a total number of 36 and 47 proteins in the 500FG and Lifelines Deep cohort respectively. In both cohorts, SNPs with MAF < 5% were removed before QTL mapping, resulting in approximately 5 M SNPs in each cohort. Finally, we used the summary statistics of pQTLs mapped in each cohort for meta-analysis. Proteins that were measured in at least 85% of all samples in both cohorts were used for meta-analysis, resulting in 35 proteins. [file Image_1.jpeg]

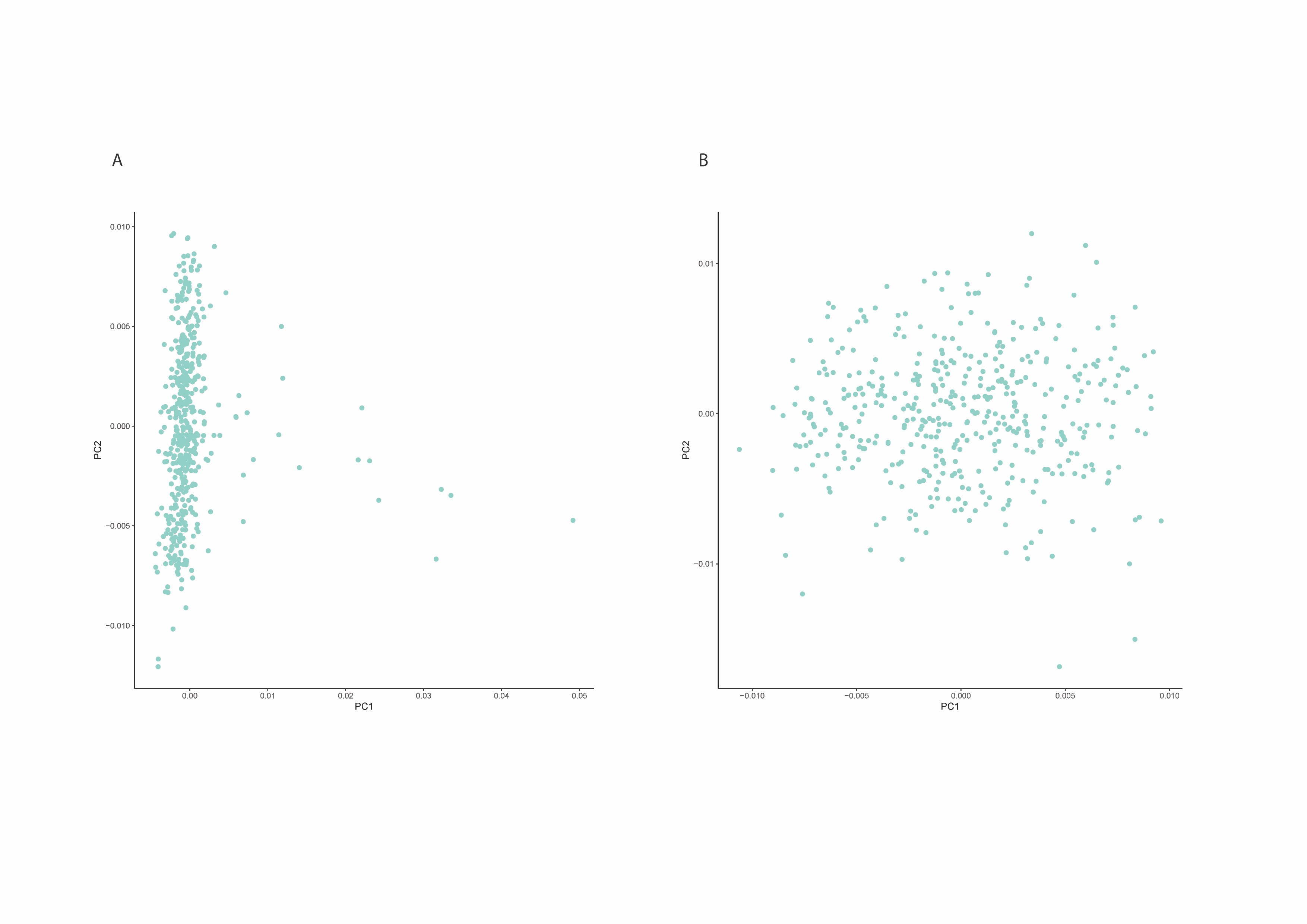

Supplement: Supplementary Figure 2 — Multidimensional scaling (MDS) analysis was performed on the 500FG cohort to identify ethnic outliers. (A) MDS plot before removing ethnic outliers and (B) after removing ethnic outliers. As genetic outliers were identified as those samples with PC1 and/or PC2 three standard deviations above or below the mean of PC1 and/or PC2. [file Image_2.jpeg]

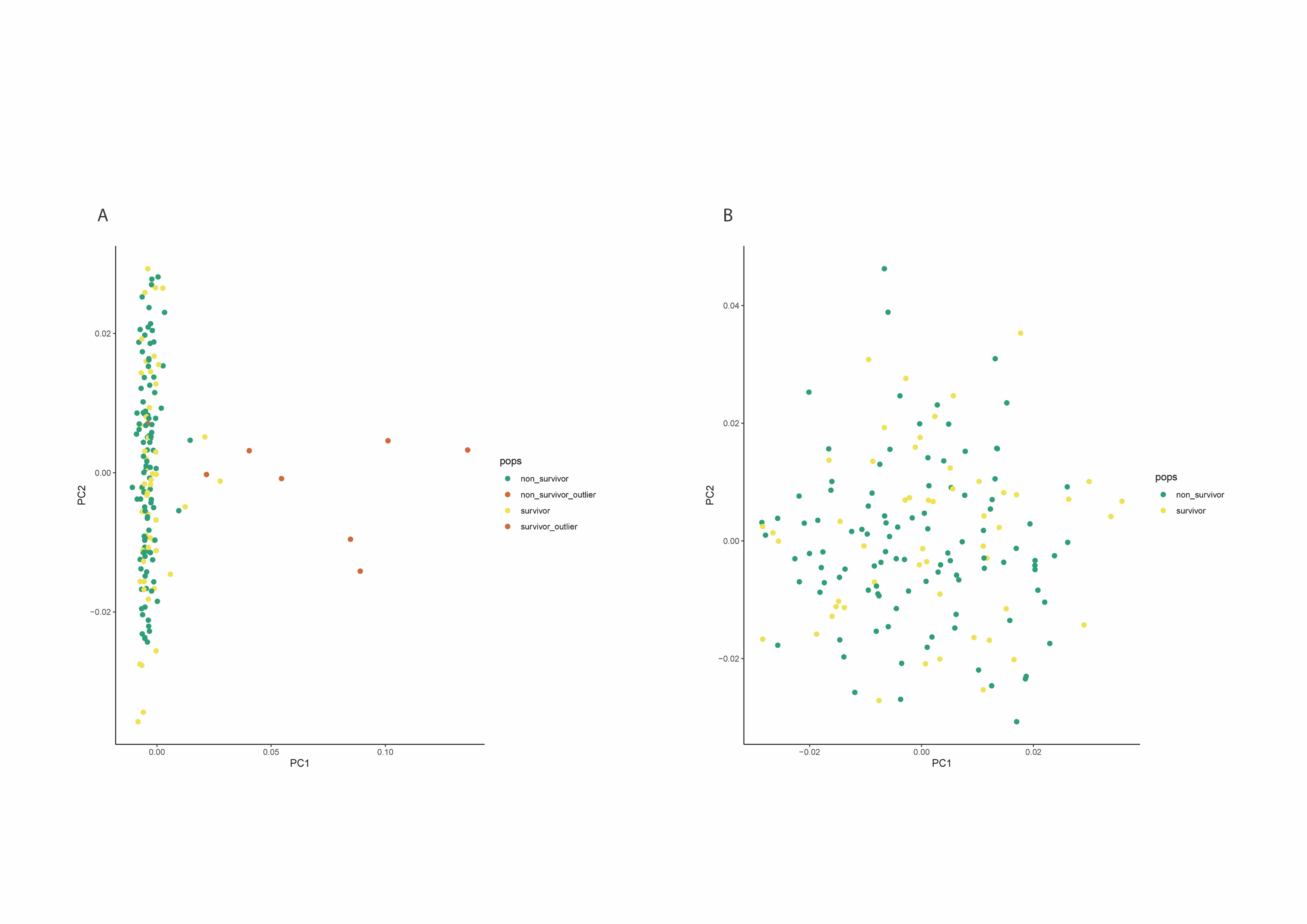

Supplement: Supplementary Figure 3 — Multidimensional scaling (MDS) analysis was performed on candidaemia patients grouped in non-survivors (n = 115 individuals, green dots) and survivors (n = 56 individuals, yellow dots). (A) MDS plot highlighting the ethnic outliers. As ethnic outliers were identified as those samples with PC1 and/or PC2 three standard deviations above or below the mean of PC1 and/or PC2 (8 individuals, orange dots). (B) MDS plot after removing the ethnic outliers. [file Image_3.jpeg]

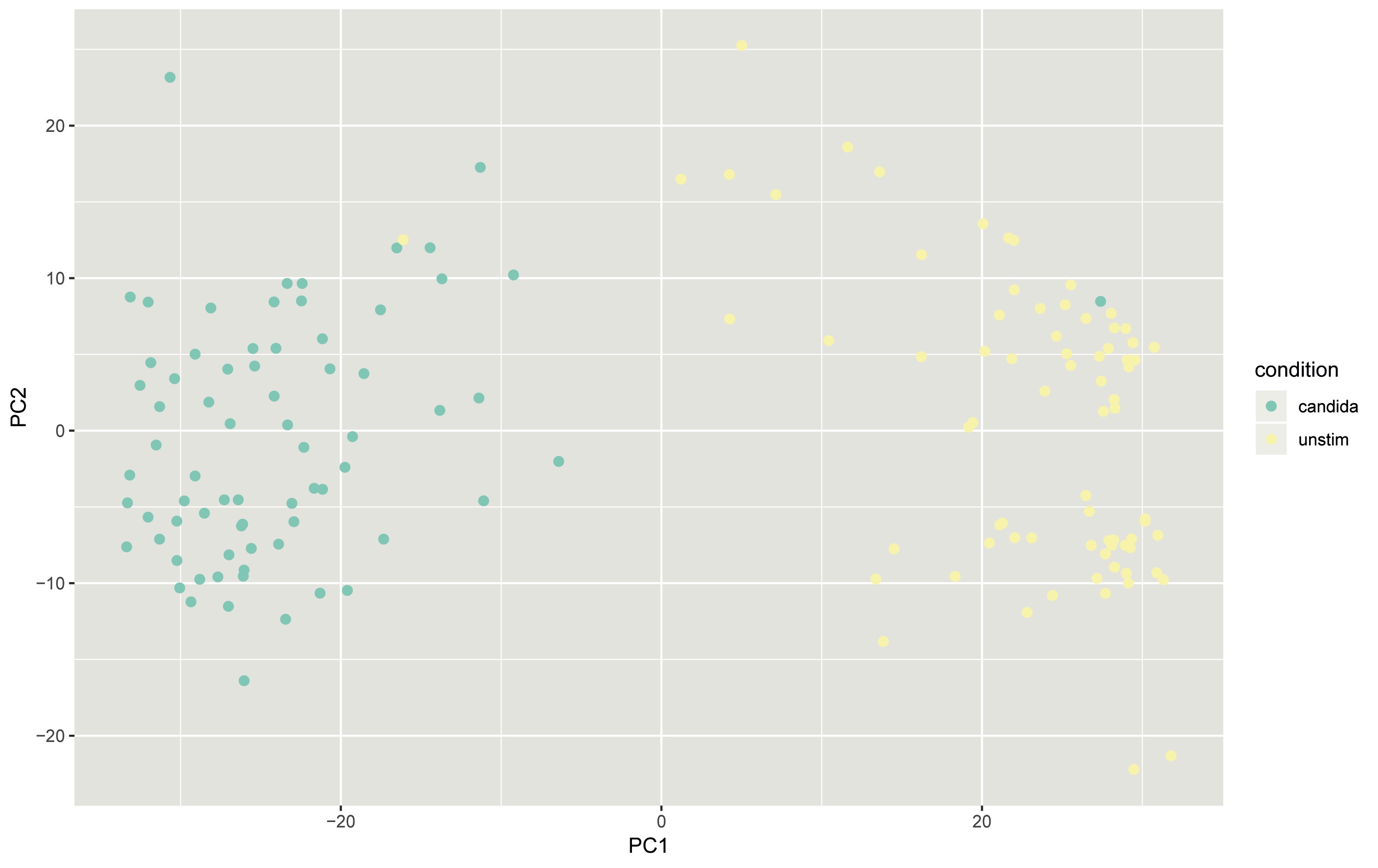

Supplement: Supplementary Figure 4 — Principal component analysis (PCA) for all genes measured in PBMC samples stimulated with C. albicans (green dots) versus RPMI 1640 medium-stimulated samples (yellow dots) in the GoNL cohort. [file Image_4.jpeg]

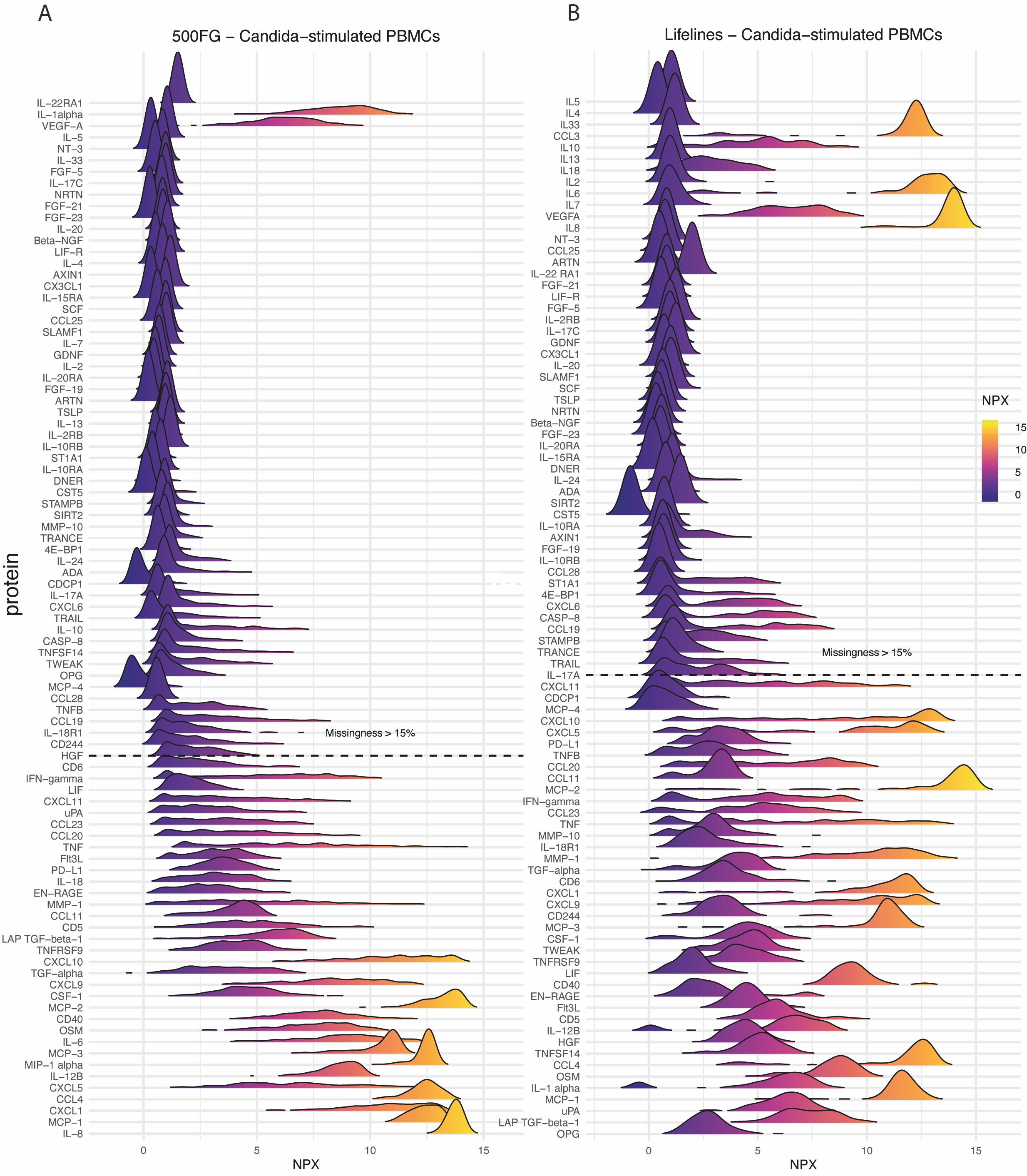

Supplement: Supplementary Figure 5 — Distribution plots of proteins measured in supernatant of Candida-stimulated PBMC samples from the (A) 500FG cohort and (B) Lifelines Deep cohort. The total number of proteins that were measured in at least 85% of samples was 36 proteins and 47 in the 500FG and Lifelines Deep cohort respectively. The total number of shared proteins between the two cohorts that were measured in at least 85% of the samples and used for meta-analysis was 35 proteins. Axis x represents the protein expression levels as NPX values (in Log2 scale). [file Image_5.jpeg]

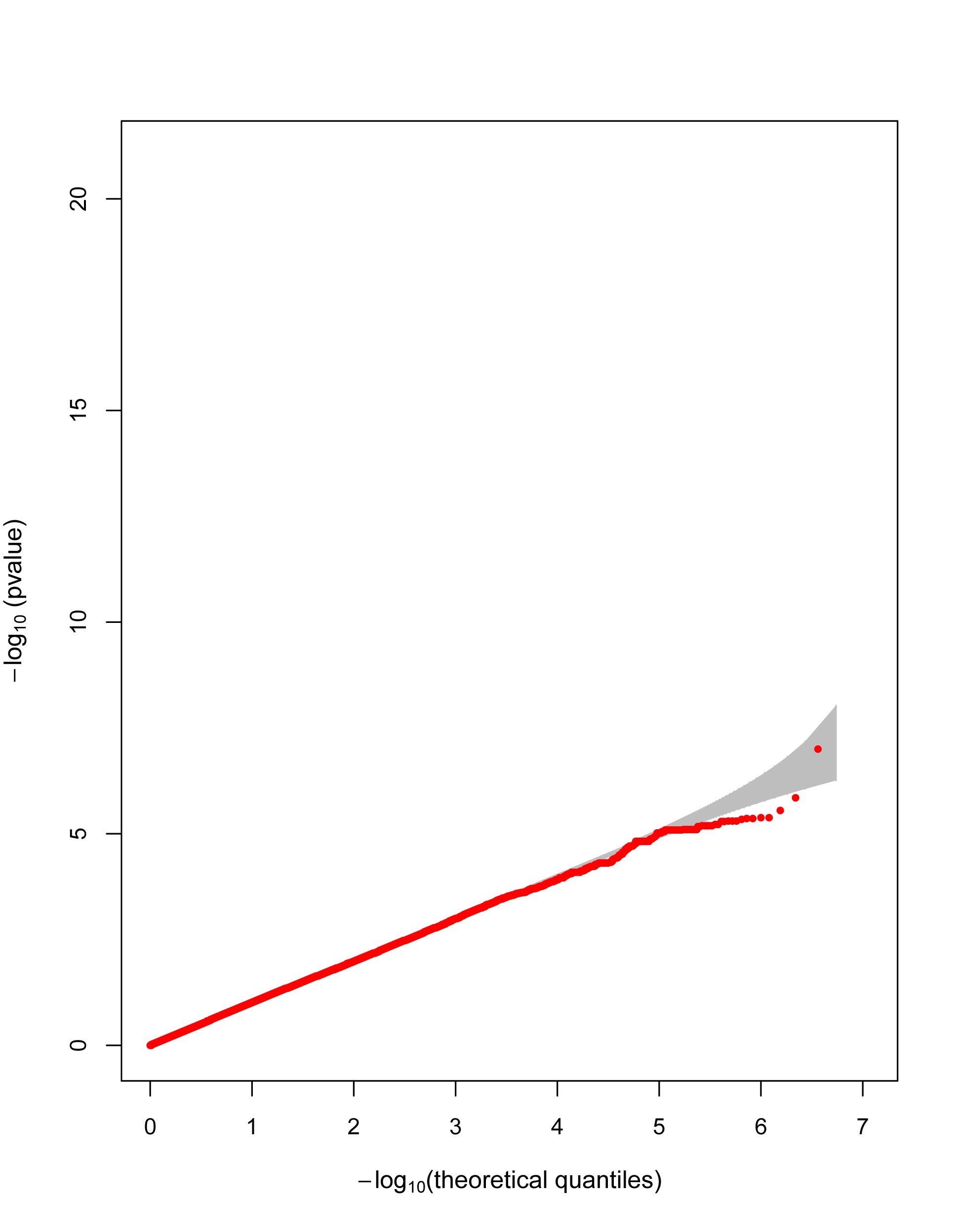

Supplement: Supplementary Figure 6 — Quantile-Quantile (QQ) plot of association P-values for all SNPs on the genome-wide genotyping array (Black dots). The genetic inflation factor λ for all SNPs was 1.03, indicating no population stratification in the survival cohort of candidaemia patients. [file Image_6.jpeg]

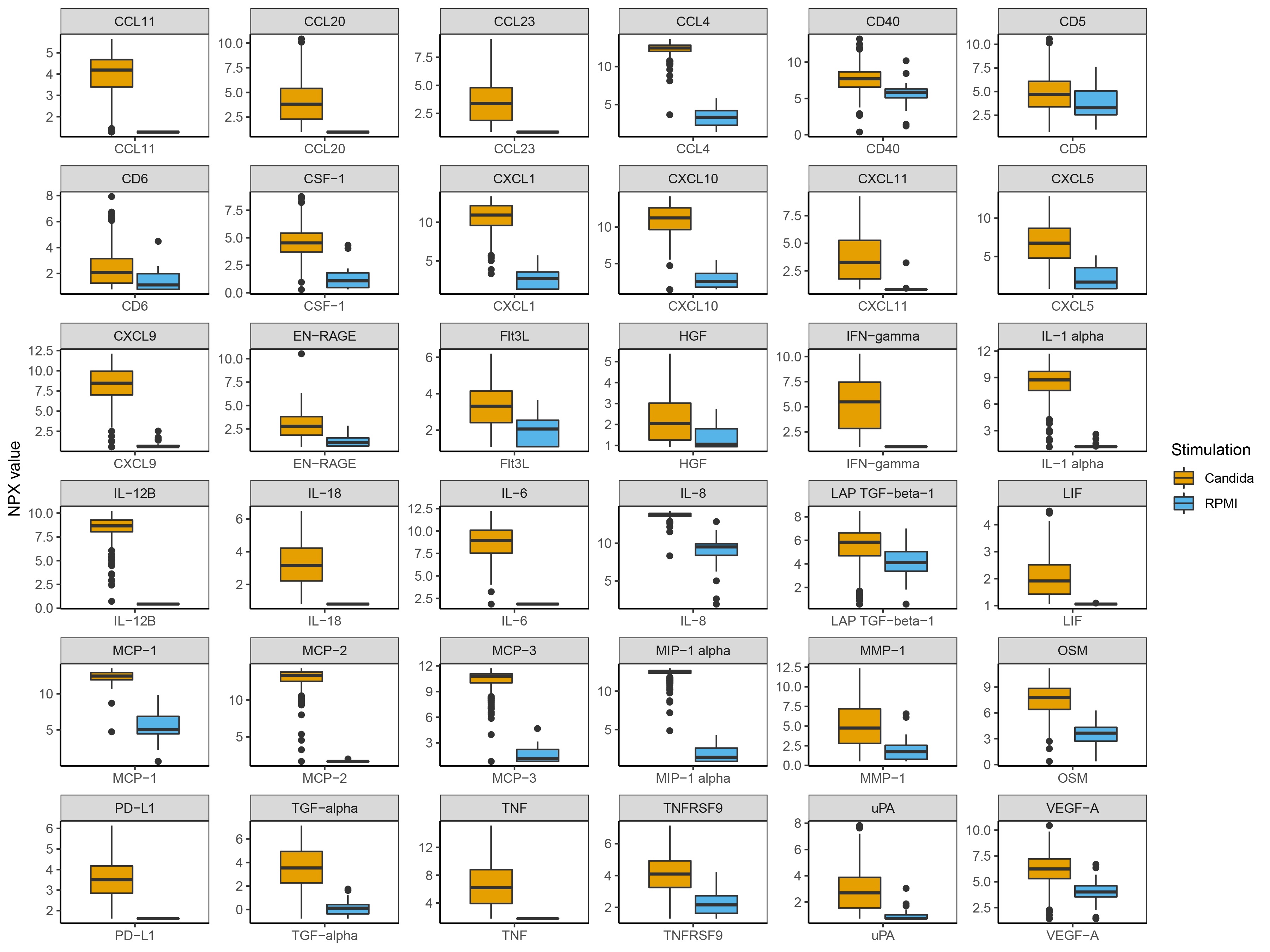

Supplement: Supplementary Figure 7 — Inflammatory proteins showed significant inter-individual differences to C. albicans stimulation compared to RPMI 1640 medium control in the 500FG cohort. [file Image_7.jpeg]

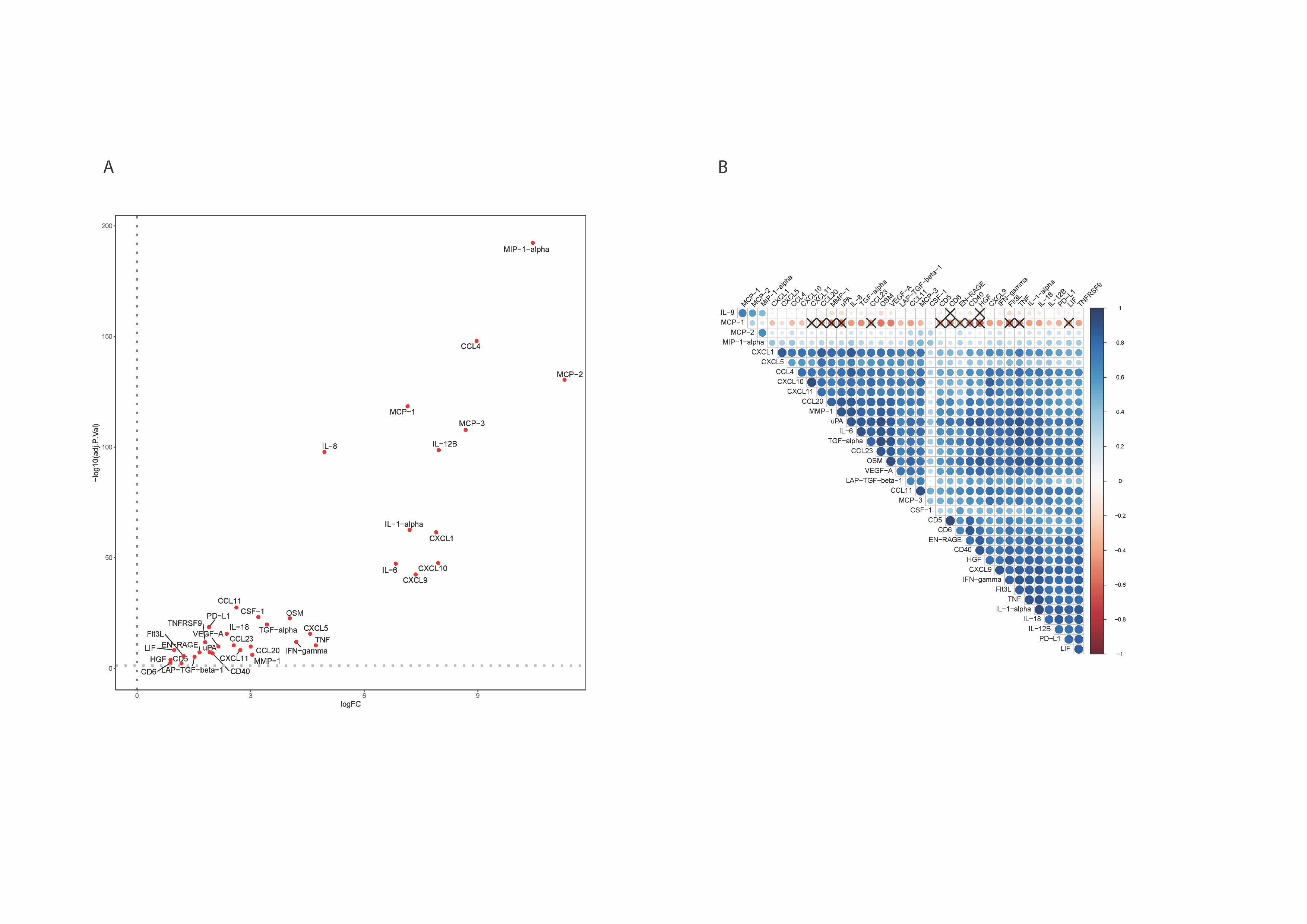

Supplement: Supplementary Figure 8 — (A) Differential expression protein analysis in Candida-stimulated versus RPMI 1640 medium-stimulated PBMC samples from the 500FG cohort. Volcano plot of proteins showing significantly differentially expressed proteins with adjusted P < 0.05 (red). Benjamini-Hochberg method used to correct for multiple testing, and adjusted P values < 0.05 were considered significant. Age and sex used as covariates. (B) Unsupervised hierarchical clustering of inflammatory proteins that were measured in at least 85% of PBMC samples stimulated with C. albicans yeast. Spearman’s correlation was used as the measure of similarity. The blue color depicts the strong positive correlation whereas the red color indicates the strong negative correlation. Correlations between pairs of proteins that show a P value > 0.01 were crossed on the plot. [file Image_8.jpeg]
